# Acacetin Ameliorates Experimental Colitis in Mice via Inhibiting Macrophage Inflammatory Response and Regulating the Composition of Gut Microbiota

**DOI:** 10.3389/fphys.2020.577237

**Published:** 2021-01-18

**Authors:** Junyu Ren, Bei Yue, Hao Wang, Beibei Zhang, Xiaoping Luo, Zhilun Yu, Jing Zhang, Yijing Ren, Sridhar Mani, Zhengtao Wang, Wei Dou

**Affiliations:** ^1^The MOE key Laboratory of Standardization of Chinese Medicines, Shanghai Key Laboratory of Compound Chinese Medicines, and the SATCM key Laboratory of New Resources and Quality Evaluation of Chinese Medicines, Institute of Chinese Materia Medica, Shanghai University of Traditional Chinese Medicine (SHUTCM), Shanghai, China; ^2^Department of Medicine and Genetics, Albert Einstein College of Medicine, The Bronx, NY, United States

**Keywords:** ulcerative colitis, gut microbiota, inflammatory mediators, dextran sulfate sodium, acacetin

## Abstract

Acacetin, a natural dietary flavonoid abundantly found in acacia honey and citrus fruits, reportedly exerts several biological effects, such as anti-tumor, anti-inflammatory, and anti-oxidative effects. However, the effects of acacetin on intestinal inflammation remain unclear. We sought to investigate whether acacetin ameliorates inflammatory bowel disease (IBD) in mice with dextran sulfate sodium (DSS)-induced ulcerative colitis (UC). Our results suggest that acacetin alleviates the clinical symptoms of DSS-induced colitis, as determined by body weight loss, diarrhea, colon shortening, inflammatory infiltration, and histological injury. Further studies showed that acacetin remarkably inhibited both the macrophage inflammatory response *in vitro* and levels of inflammatory mediators in mice with colitis. In addition, some features of the gut microbiota were disordered in mice with DSS-induced colitis, as evidenced by a significant reduction in microbiota diversity and a marked shift in bacterial profiles. However, acacetin treatment improved this imbalance and restored gut microbiota to levels that were similar to those in normal mice. In conclusion, our work presents evidence that acacetin attenuates DSS-induced colitis in mice, at least in part, by inhibiting inflammation and regulating the intestinal microbiota.

## Introduction

Ulcerative colitis (UC) is a major form of inflammatory bowel disease (IBD), characterized by continuous and diffuse inflammatory lesions of the colorectal mucosa. The original distribution of UC included European and American countries. However, with the development of global industrialization, as well as changing lifestyles and the living environment, UC has gradually become a common disease worldwide. In developing countries with large populations, the incidence of UC increases each year, which causes considerable mental stress and brings an economic burden to the affected patients (Kaplan, 2015; Ananthakrishnan et al., 2018). Abdominal pain, diarrhea and mucopurulent bloody stools are the main clinical manifestations of UC, which usually first affects the rectum and then spreads to the whole colon (Sun B. et al., 2018). The etiology of UC is unclear and it is thought to be influenced by a combination of several factors, such as genetic susceptibility, impaired intestinal integrity, dysfunctional immune responses, and environmental pathogens (Zhang et al., 2014; Ramos and Papadakis, 2019). Several treatments have been developed for UC, including glucocorticoids, sulfasalazine, and immunosuppressive drugs (Hu et al., 2019). However, the clinical application of these drugs in long-term treatment is limited by their adverse effects and the high recurrence rates observed, which necessitates the development of novel therapies or complementary and alternative medicine for IBD.

In the past few decades, an increasing number of studies have suggested that alterations in the composition of gut microbiota can play key roles in the pathogenesis of UC. Patients with UC are often characterized by a damaged intestinal mucosal barrier, reduced microbial diversity, and disrupted balance of “harmful” and “protective” bacteria in the intestine (Kostic et al., 2014; Kiely et al., 2018). In particular, the composition of three major bacterial phyla in the gut microbiota are reportedly disturbed in patients with UC, as manifested by a reduced proportion of Firmicutes and Bacteroidetes, and an increased proportion of Proteobacteria (Smits et al., 2013; Matsuoka and Kanai, 2015). The transplantation of fecal bacteria and probiotic therapy in patients with UC are reportedly effective therapeutic approaches, and have shown promising outcomes in clinical practice (Zuo and Ng, 2018; Burrello et al., 2019; Khan et al., 2019). Furthermore, the disturbance of intestinal flora may serve as a major factor in the development of UC. Disordered gut microbiota may damage the intestinal barrier, promote intestinal permeability, activate the immune system, and ultimately contribute to the occurrence and progression of UC (Ott, 2004; Kostic et al., 2014; Kiely et al., 2018).

Epidemiological evidence suggests that increased dietary intake of flavonoid-rich fruits and vegetables is associated with a lower risk of IBD (Hu et al., 2019). Flavonoid compounds comprise a large family of hydroxylated polyphenolic molecules that are abundant in plants and have potential therapeutic potency in IBD (Salaritabar et al., 2017). Acacetin (5,7-dihydroxy-4’-methoxyflavone), a flavone widely recognized as an important bioactive constituent of acacia honey and citrus fruits, reportedly exerts protective effects on the gastrointestinal tract (Zhang et al., 2017; Yin et al., 2019). Acacetin can be isolated from the *Saussurea involucrata* plant (Liou et al., 2017), damiana (*Turnera diffusa*), and black locust (*Robinia pseudoacacia*) (Punia et al., 2017; Prasad et al., 2020). In addition, acacetin reportedly has various pharmacological activities, including antioxidant, anti-inflammatory, and anti-tumor activities (Xiao et al., 2019). However, its effects in UC and the associated underlying mechanisms remain unknown. In this study, we present evidence that acacetin could relieve UC-associated symptoms in mice, and show that the attenuated effects were mediated, at least in part, by inhibiting the macrophage inflammatory response and regulating the composition of gut microbiota.

## Materials and Methods

Bone marrow-derived macrophages (BMDMs) culture and treatment, and immunohistochemical staining assay details are mentioned in Supplemental Methods.

### Materials

Acacetin (C_16_H_12_O_5_, molecular weight: 284.26, high-performance liquid chromatography purity ≥98%) was purchased from Chengdu Biopurify Phytochemicals Ltd. Dextran sulfate sodium (DSS) (molecular mass, 36–50 kDa) was purchased from MP Biochemical (Irvine, CA, United States). RAW264.7 mouse macrophage cells were purchased from the American Type Culture Collection (ATCC, Manassas, VA, United States). Dulbecco’s modified Eagle’s medium (DMEM), 100 U/ml penicillin/streptomycin and fetal bovine serum (FBS) were purchased from Gibco BRL (Grand Island, NY, United States). Diethylpyrocarbonate-treated water, lipopolysaccharide (LPS), dimethyl sulfoxide, paraformaldehyde, and diaminobenzidine were obtained from Sigma-Aldrich (Shanghai, China). The enhanced chemiluminescence (ECL) detection kit was obtained from Millipore (Billerica, MA, United States). Rabbit antibodies to inducible nitric oxide synthase (iNOS) (#13120), cyclooxygenase-2 (COX-2) (#12282), tumor necrosis factor alpha (TNF-α) (#3707S), and β-actin (#4970) were obtained from Cell Signaling Technology (Danvers, MA, United States). Rabbit antibodies to interleukin 6 (IL-6) (ab83339) were obtained from Abcam (Cambridge, MA, United States). Trizol and the SuperScript II Reverse Transcriptase kit were purchased from Thermo Scientific Inc., (Waltham, MA, United States). The SYBR Premix ExTaq Mix was purchased from Takara Biotechnology (Shiga, Japan). The cell counting kit 8 (CCK-8) assay kit was purchased from Meilun Biological Technology Co., Ltd. All other reagents were obtained from Thermo Fisher Scientific (Waltham, MA, United States).

### Mice and DSS-Induced Colitis

Healthy, female C57BL/6 mice (20 ± 2 g), 6–8 weeks old, were purchased from Shanghai Laboratory Animal Center, they were maintained under specific pathogen-free conditions at a fixed temperature range of 23–25°C and relative humidity of 55 ± 10%, with a 12-h light/dark cycle. All mice were allowed to acclimatize for 7 days after arrival and supplied *ad libitum* a standard dry diet and tap water. All animal study protocols were approved and carried out in accordance with the principles of the declaration recommendations of the Animal Experimentation Ethics Committee at Shanghai University of Traditional Chinese Medicine (Animal license key: PZSHUTCM190315022).

As described previously (Dou et al., 2014), the experiment lasted for 9 days. Acute experimental colitis was induced in mice by administering drinking water containing 3.5% (w/vol) DSS for 7 days, while control mice received drinking water only. Acacetin was dissolved in 0.5% methylcellulose at dosages of 50 and 150 mg/kg. Mice were randomly distributed into the following five groups (*n* = 10 mice per group): Group 1 was treated as vehicle controls, which were administered 100 μL of 0.5% (wt/vol) methylcellulose by oral gavage once per day; Group 2 was the acacetin group, which was administered acacetin at a daily dose of 150 mg/kg throughout the experiment; Group 3 was the DSS group, which was administered 3.5% DSS in the drinking water from day 3 to day 9 and 100 μL of 0.5% (wt/vol) methylcellulose by oral gavage once per day; Groups 4 and 5 received acacetin at doses of 50 and 150 mg/kg/day per body weight, respectively, by oral gavage from day 1 to day 9.

### Histological Assessment of Colitis

Mice were monitored and daily records were maintained for changes in body weight, diarrhea, and bloody stools. Mice were sacrificed under anesthesia on day 9, and 4 h after the last gavage. The entire colon was then removed and its length was measured. The distal colon was resected, fixed in 10% formaldehyde overnight, and embedded in paraffin. The paraffin-embedded colon sections were stained with hematoxylin and eosin for histological evaluation. Histological injury was evaluated blindly by a combination of inflammatory cell infiltration (score 0–3) and mucosal damage (score 0–3), as described previously (Zhang et al., 2015).

### Fecal Microbiota 16S rRNA Gene Sequencing

Mice feces were collected and stored at −80°C. Total genomic DNA was extracted from fecal samples using the E.Z.N.A.^®^ Soil DNA kit (Omega Bio-tek, GA, United States) according to the manufacturer’s protocols. The concentration and quality of DNA were checked using the NanoDrop 2000 UV-Vis spectrophotometer (Thermo Scientific, Wilmington, DE, United States). The V3–V4 regions of the bacterial 16S rRNA were amplified with the universal primers, 338F (5′-ACT CCTACGGGAGGCAGCAG-3′) and 806R (5′-GGACTACH VGGGTWTCTAAT-3′) using a thermocycler PCR system (GeneAmp 9700 ABI, Carlsbad, CA, United States). The protocol included amplification at 95°C for 3 min in the first instance, followed by 27 cycles (denaturation at 95°C for 30 s, annealing at 55°C for 30 s, and elongation at 72°C for 45 s), and a final extension (72°C, 10 min). Sequencing and data analysis were then performed on the Illumina MiSeq platform (Illumina, San Diego, CA, United States) according to the standard protocol of Majorbio Bio-Pharm Technology Co., Ltd (Majorbio, Shanghai, China). Sequence similarities of samples ≥97% were classified as the same operational taxonomic units (OTUs). The taxonomy of each 16S rRNA gene sequence was analyzed via the RDP Classifier algorithm against the Silva (SSU123) 16S rRNA database using a 70% confidence threshold.

### Immunoblotting Analysis

Proteins extracted from colon tissues (1–1.5 cm proximal to the anus) and cultured cells were homogenized and lysed in cold radioimmunoprecipitation assay buffer with a suitable concentration of protease and phosphatase inhibitor cocktail tablets. The homogenate was then centrifuged (4°C, 12000 *g*, 15 min) and the supernatant was collected. The total protein concentration was determined via the bicinchoninic acid assay. Total protein (30 mg) from each sample was prepared and separated using either 10% or 12% SDS-PAGE, and subsequently transferred to 0.2 μm polyvinylidene fluoride membranes. Membranes were blocked with 5% (w/v) skim milk for 2 h at 25°C, and incubated overnight with primary antibodies against iNOS, COX-2, IL-6, TNF-α, or β-actin. The membranes were then washed three times with phosphate-buffered saline with Tween^®^ for 10 min. The corresponding HRP-conjugated secondary antibody was added and membranes were further incubated for 1 h. The immunoreactive bands were observed with the aid of an ECL detection reagent and the images of blots were analyzed on a GS-700 imaging densitometer (Bio-Rad, CA, United States). The housekeeping gene, β-actin, was used as an internal control.

### Quantitative Real-Time PCR (qPCR) Analysis

All experimental procedures followed the respective kit manufacturer’s protocols. Total RNA was extracted from colon samples using the TRIzol reagent. Complementary DNA (cDNA) was reverse transcribed using the SuperScript II Reverse Transcriptase kit and 1.5 μg total RNA from each sample. Primers for the inflammatory mediators and internal reference were as follows: interleukin 1 beta (IL-1β): forward, 5′-ATTGTGGCTGTGGAGAAGAAGA-3′, reverse, 5′-TGAAG GAAAAGAAGGTG-3′; IL-6: forward, 5′-GCCTTCCCTACTTC ACAA-3′, reverse, 5′-ACAACTCTTTTCTCATTTCCAC-3′; iNOS: forward, 5′-GTCCTACACCACACCAAACT-3′, reverse, 5′-ATCTCTGCCTATCCGTCTC-3′; COX-2: forward, 5′-ACAA CAACTCCATCCTCCT-3′, reverse, 5′-GGTATTTCATCTCTCT GCTCTG-3′; TNF-α: forward, 5′-CTCTTCTCATTCCTGCT TGT-3′, reverse, 5′-GTGGTTTGTGAGTGTGAGG-3′; and β-actin: forward, 5′-GGGAAATCGTGCGTGAC-3′, reverse, 5′-AGGCTGGAAAAGAGCCT-3′. The qPCR followed the protocol of the Takara SYBR Green Master Mix Kit and was quantitatively analyzed using the ABI Prism 7900HT Sequence Detection System (Life Technologies, Carlsbad, CA, United States). The internal control was β-actin.

### Cell Culture and Cell Viability Assay

The RAW264.7 cells were cultured in Dulbecco’s modified Eagle Medium supplemented with 10% fetal bovine serum and a mixture of antibiotics (100 units/mL penicillin and streptomycin) under 5% CO_2_ at 37°C. The RAW264.7 cells (2 × 10^5^/well) were seeded in a 96-well plate overnight. Cells were subsequently incubated with a wide dose range of acacetin (0, 5, 10, 15, 20, 25, 30, 35, 40, and 45 μmol/L) for 24 h. Cell viability was determined using a CCK-8 assay kit. A volume of 10 μL of the CCK-8 solution was added to each well, which was then incubated for 30 min at 37°C. Absorbance was measured at 450 nm using a microplate reader.

### Measurement of Nitric Oxide (NO) Production

The RAW264.7 cells were seeded in a 96-well plate at a density of 1 × 10^5^ cells/well, and then incubated with different doses of acacetin (0, 5, 10, 15, 20, 25, 30, and 40 μmol/L) for 2 h, prior to stimulation with LPS (1 μg/mL) for an additional 24 h. The cell culture media were collected and the Griess reagent was used to detect NO production. Absorbance was measured at 540 nm using a microplate reader.

### Statistics

All data were presented as the mean ± SEM. Differences between groups were analyzed with one-way analysis of variance using the GraphPad Prism 7 software (GraphPad Software, La Jolla, CA, United States). All *p*-values < 0.05 (two-sided) were considered significant. All 16S rDNA sequencing data were analyzed on the Majorbio I-Sanger Cloud Platform online (www.i-sanger.com).

## Results

### Acacetin Ameliorated DSS-Induced Acute Colitis in Mice

**FIGURE 1:**
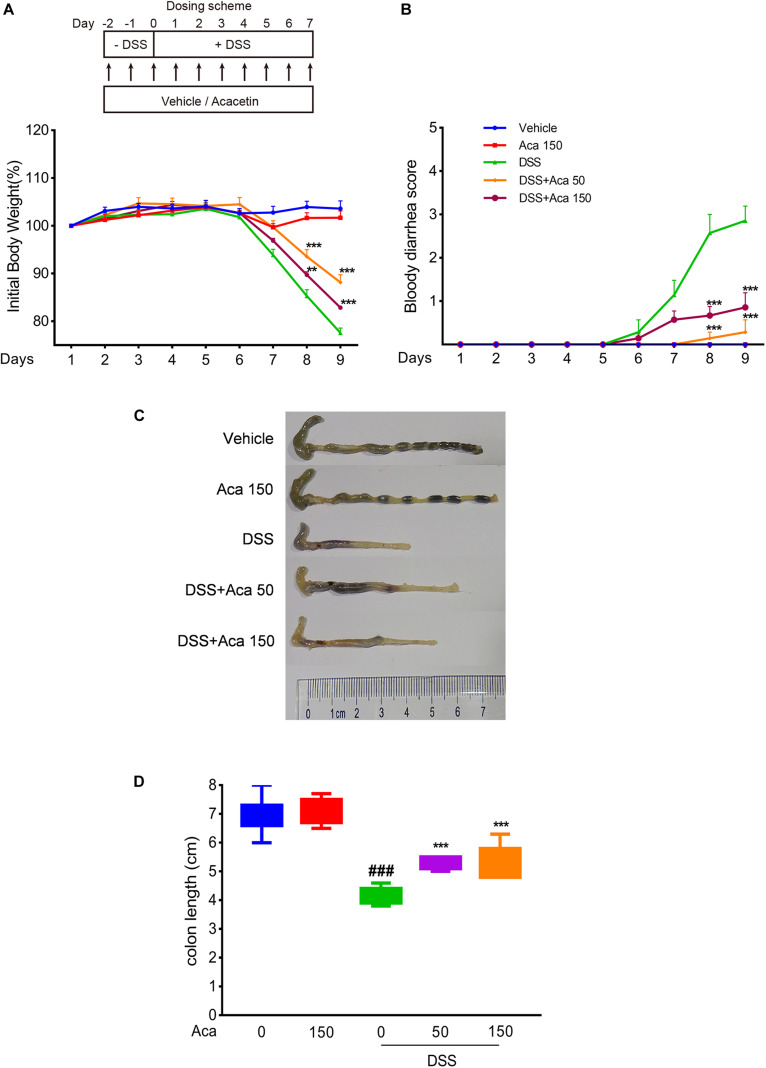
Acacetin ameliorated weight loss, bloody diarrhea, and colon shortening in dextran sulfate sodium (DSS)-treated mice. **(A)** Mice body weight changes after induction of colitis by DSS. The data were plotted as a percentage of the weight at baseline. **(B)** Bloody diarrhea score. The data were plotted as scores for bloody diarrhea following DSS treatment at different time points. Macroscopic observation **(C)** and assessment of colon shortening **(D)** after DSS treatment. Data were expressed as the mean ± SEM (*n* = 7 mice in each group). **p < 0.005, ***p < 0.001 vs. the DSS group; ^###^*p* < 0.001 vs. the vehicle group.

**FIGURE 2:**
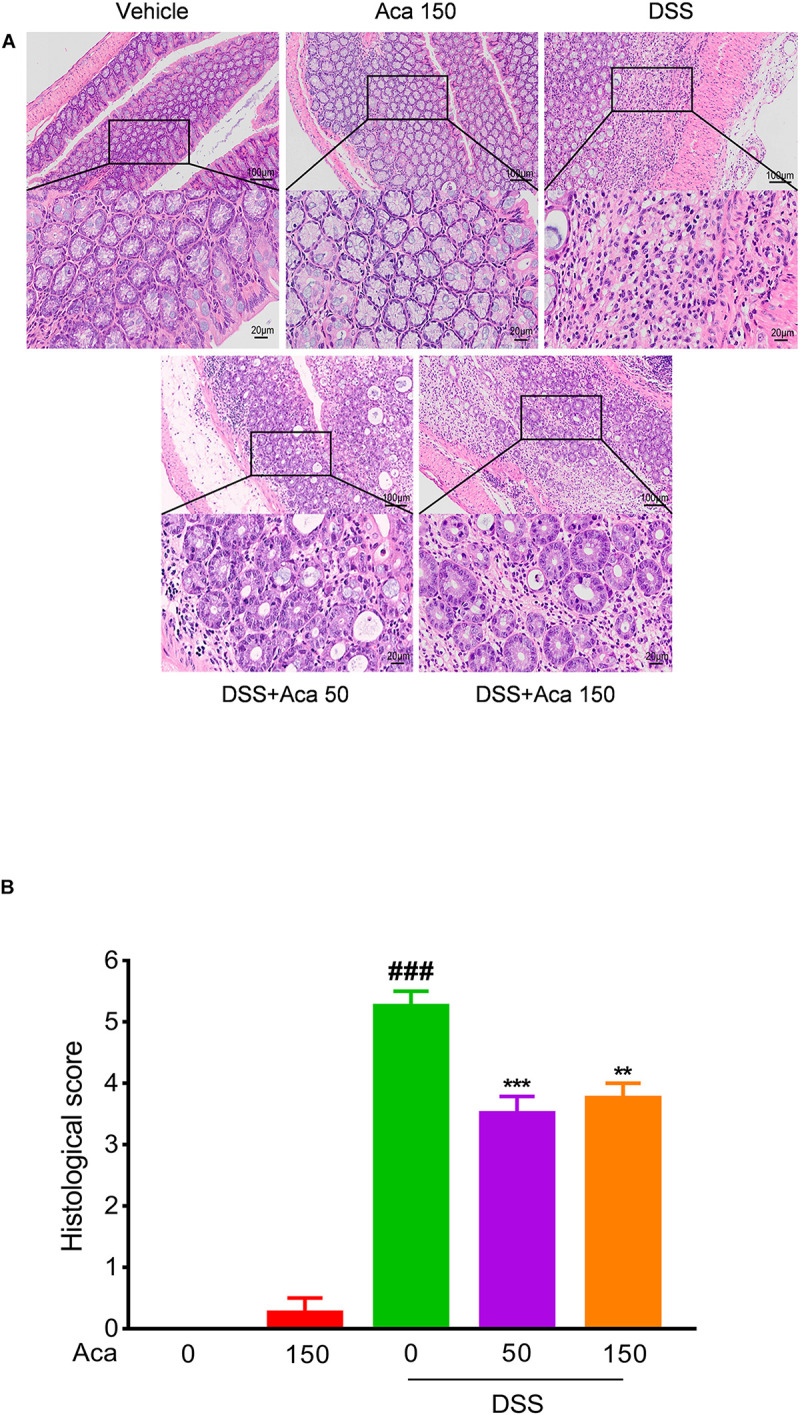
Acacetin ameliorated inflammatory infiltration and histopathological damage in dextran sulfate sodium (DSS)-treated mice. Representative hematoxylin and eosin-stained colon sections **(A)** and histological score **(B)**. The scale bar corresponds to 100 μm or 20 μm. Data were presented as the mean ± SEM (*n* = 7 mice per group). **p < 0.01, ***p < 0.001 vs. the DSS group; ^###^*p* < 0.001 vs. the vehicle group.

The mice with DSS-induced colitis showed significant weight loss and diarrhea, while mice treated with acacetin (50 or 150 mg/kg) showed significantly milder colitis symptoms (Figures 1A,B). As with other indexes, such as body weight, diarrhea, and colon length, the length of the colon in the DSS group was significantly shortened compared with the other four groups. After the administration of acacetin (50 or 150 mg/kg), the reduction in colon length was reversed (Figures 1C,D). No significant differences in body weight and colon length were observed between the control group and acacetin treatment group (Figures 1A–D). In addition, no signs of marked inflammation were observed in the intestines of the control group or acacetin (alone) treatment group, based on macroscopic histopathological analysis. However, intestinal tissue injury was apparent in the colon of the DSS model group, which showed severe epithelial damage, abnormal crypt structures, and extensive neutrophilic infiltration. Acacetin (50 or 150 mg/kg) treatment alleviated the loss of mucosal architecture, ulcerations, and inflammatory infiltration (Figures 2A,B). Specifically, acacetin (50 mg/kg) produced the best phenotypes, and the 50 mg/kg acacetin treatment group was thus applied for subsequent experimental analyses. Collectively, these results suggest that acacetin could ameliorate the symptoms of DSS-induced colitis.

### Acacetin Inhibited the Production of Inflammatory Mediators in Macrophages

**FIGURE 3:**
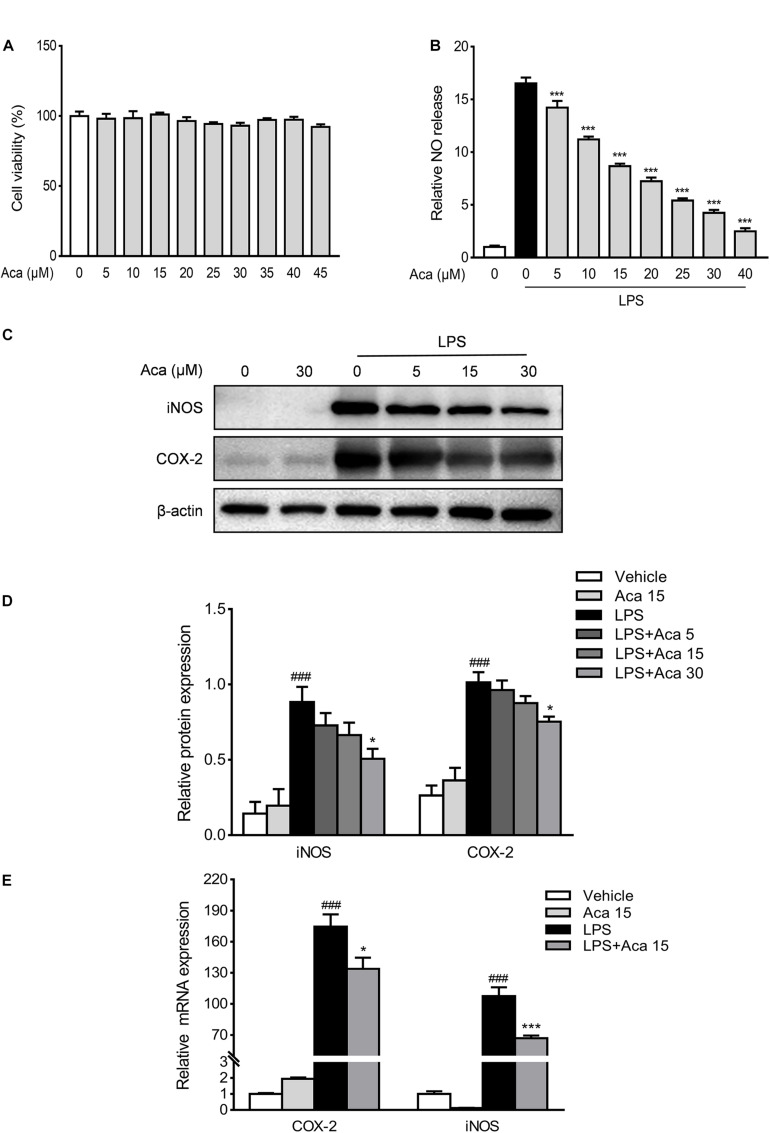
Acacetin inhibited the expression of inflammatory mediators *in vitro*. Effects of acacetin on cell viability **(A)** and the secretion of nitric oxide (NO) **(B)** in lipopolysaccharide (LPS)-induced RAW264.7 cells. **(C)** Protein expression levels of LPS-induced RAW264.7 cells, including inducible nitric oxide synthase (iNOS) and cyclooxygenase-2 (COX-2), were determined by western blot analysis. **(D)** Quantification of protein expression was determined by densitometric analysis of the blots. Expression was normalized to β-actin. **(E)** Effects of acacetin on the expression of proinflammatory mediator mRNA in LPS-induced RAW264.7 cells. The mRNA expression of COX-2 and iNOS were determined by qPCR. All expressions were normalized to β-actin. Data are presented as the mean ± SEM (*n* = 3). *p < 0.05, ***p < 0.001 vs. the DSS group; ^###^*p* < 0.001 vs. the vehicle group.

Macrophages are the major source of proinflammatory mediators when inflammation occurs in the intestine (Yan et al., 2018). Therefore, we used RAW264.7 mouse macrophages to evaluate the anti-inflammatory effects of acacetin *in vitro*. We used the CCK-8 kit to test the cytotoxicity of acacetin on RAW264.7 cells. The results showed that acacetin was almost non-cytotoxic at dosages up to 45 μmol/L (Figure 3A). The iNOS in macrophages can activate the secretion of NO in inflammatory states (Jiang et al., 2020). Our results show that acacetin treatment can reduce the production of NO, as well as the protein levels of iNOS and COX-2 in LPS-induced RAW267.4 cells in a dose-dependent manner (Figures 3B–D). We quantified the mRNA expression of iNOS and COX-2 in LPS-stimulated macrophages. Acacetin treatment was found to attenuate the mRNA levels of iNOS and COX-2 in LPS-induced RAW264.7 cells (Figure 3E). In addition, acacetin treatment also decreased the secretion of NO, as well as the protein level of iNOS in IFN-γ and LPS induced bone marrow-derived macrophages (BMDMs) (Supplementary Figure 1). Hence, acacetin was able to inhibit the production of inflammatory mediators in RAW264.7 cells and BMDM cells.

### Acacetin Decreased Macrophage Infiltration and the Levels of Inflammatory Mediators in Mice With Colitis

**FIGURE 4:**
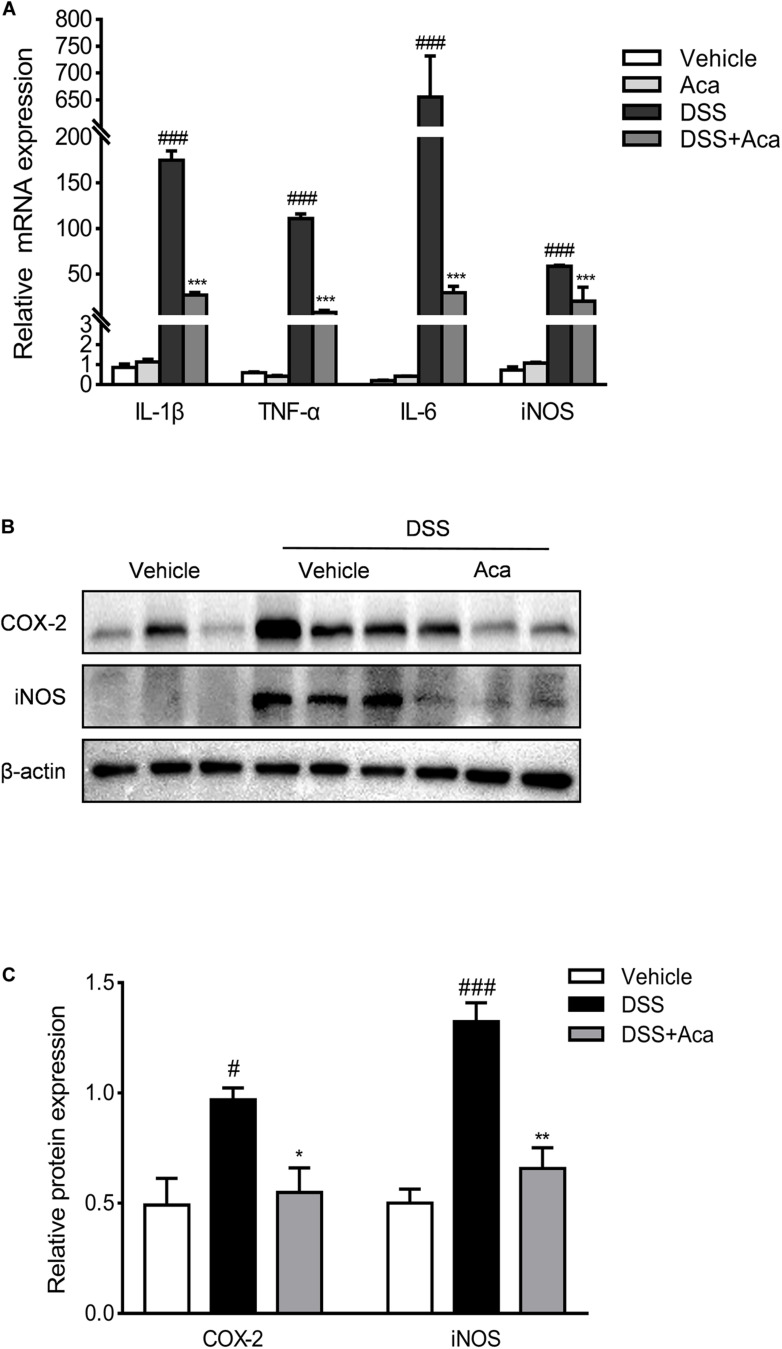
Acacetin inhibited the expression of inflammatory mediators *in vivo*. **(A)** Effects of acacetin on proinflammatory mediator gene expression in colon tissue of dextran sulfate sodium (DSS)-treated mice. The mRNA expression of interleukin 1 beta (IL-1β), tumor necrosis factor alpha (TNF-α), interleukin 6 (IL-6), and inducible nitric oxide synthase (iNOS) in colon samples were determined by qPCR. All expressions were normalized to β-actin. **(B)** Effects of acacetin on the inflammatory mediator protein expression of cyclooxygenase-2 (COX-2) and iNOS in colon tissue of DSS-treated mice. **(C)** Quantification of protein expression was performed by densitometric analysis of the blots. Expression was normalized to β-actin. Data are expressed as the mean ± SEM (*n* = 3). *p < 0.05, **p < 0.005, ***p < 0.001 vs. the DSS group; ^#^*p* < 0.05, ^###^*p* < 0.001 vs. the vehicle group.

To assess the potential innate immune influence of acacetin on DSS-induced colitis, we examined the macrophage infiltration by immunohistochemical staining of the macrophage markers F4/80. We found that the infiltration of macrophages expressing F4/80 in colon tissue was significantly decreased by acacetin treatment compared with colitis mice (Supplementary Figure 2). Several studies suggest that COX-2, iNOS, IL-6, and TNF-α are important proinflammatory mediators associated with the acute phase of IBD (Papadakis and Targan, 2000; Neurath, 2014). Therefore, we evaluated the effects of acacetin on the production of inflammatory mediators *in vivo*. As illustrated in Figure 4A, the mRNA levels of IL-1β, TNF-α, iNOS, IL-6, and iNOS were markedly increased in mice with DSS-induced colitis. However, acacetin treatment significantly reduced the mRNA expression of inflammatory mediators. On the other hand, immunoblots showed that DSS treatment led to a marked increase in the protein expression of COX-2 and iNOS in colonic tissue. However, the administration of acacetin markedly reduced the expression of COX-2 and iNOS (Figures 4B,C). These results suggest that acacetin could reduce the production of inflammatory mediators in mice with DSS-induced colitis.

### Acacetin Regulated the Gut Microbiota Composition

**FIGURE 5:**
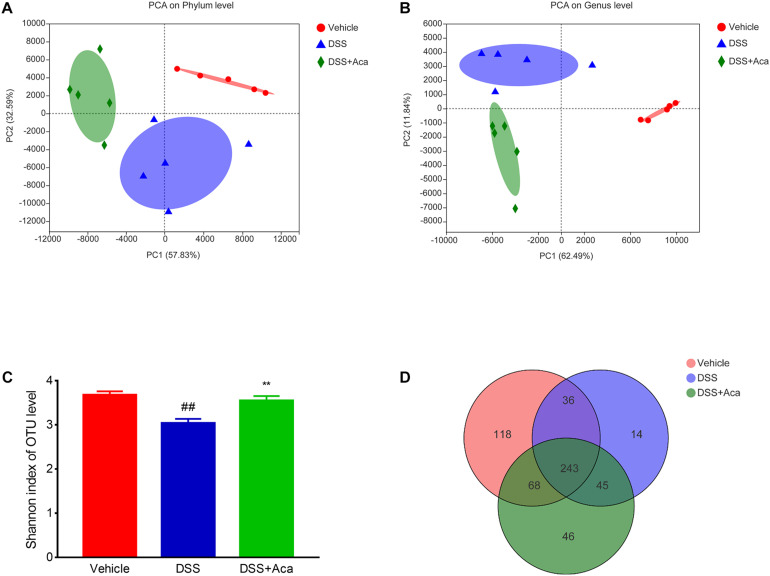
Acacetin regulated the composition of gut microbiota in mice treated with dextran sulfate sodium (DSS). **(A)** Principal component analysis (PCA) graphical results at the phylum level. **(B)** Graphical results of PCA at the genus level. **(C)** Index-group difference test of the Shannon index in sample hierarchical cluster tree, alpha diversity. Student’s *t*-test. Classification level: operational taxonomic unit (OTU). Data are presented as the mean (*n* = 5 mice per group). **(D)** Venn diagram indicates the differential numbers of OTUs in each group. **p < 0.01 vs. the DSS group; ^##^*p* < 0.01 vs. the vehicle group.

To investigate the possible effects of acacetin on DSS-induced changes in the composition of intestinal microbiota, we analyzed fecal samples through 16S rRNA sequencing. The principal component analysis showed a noticeably separate cluster between the DSS group and the control group. A similar phenomenon was observed in comparisons of the DSS group and DSS + acacetin group (Figures 5A–D). In addition, acacetin treatment recovered the community diversity and significantly shifted the gut microbiota structure in the PC1 direction in mice with DSS-induced colitis. The Shannon index revealed that DSS treatment resulted in a significant decline in intestinal microbial community diversity, compared with the control or acacetin (alone) treatment groups (Figure 5B).

**FIGURE 6:**
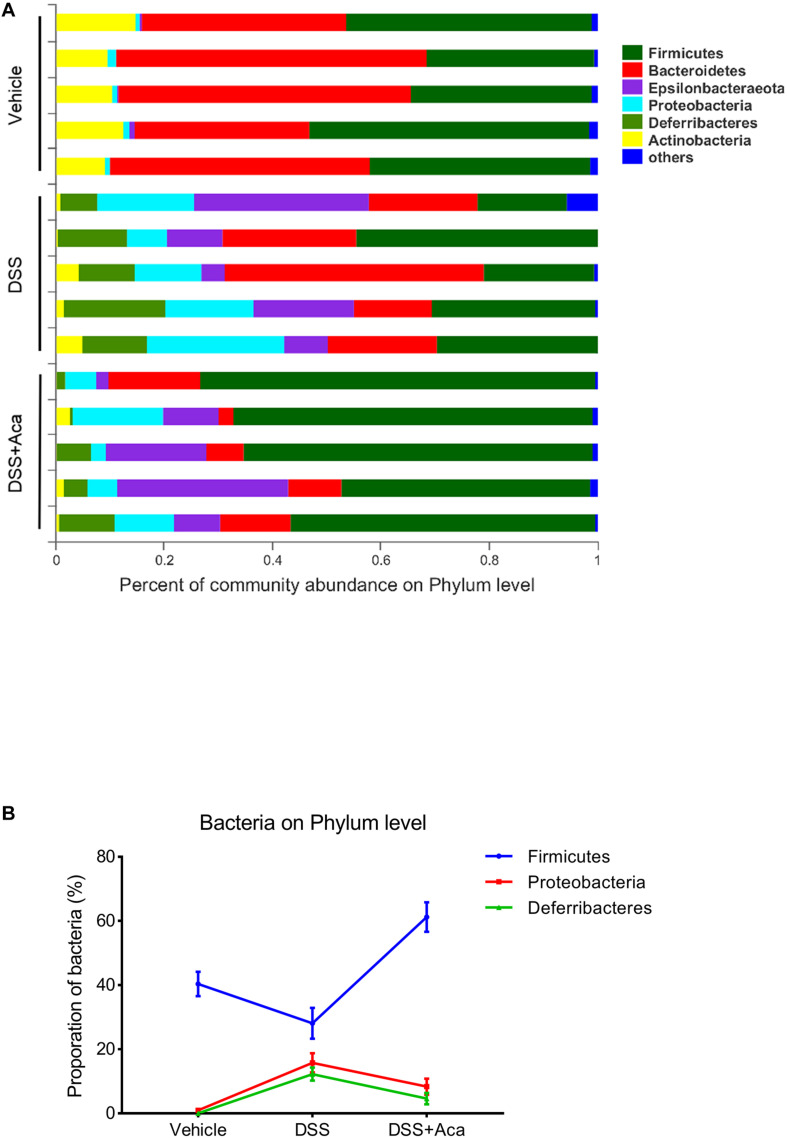
Acacetin regulated the composition and abundance of gut microbiota at the phylum level. **(A)** Proportion of dominant phylum communities in each group of samples. Less than 10% of the phylum communities were merged with others. **(B)** Distribution of the three dominant phyla (Firmicutes, Proteobacteria, and Deferribacteres) in each group. Data are presented as the mean ± SEM (*n* = 5 mice per group).

Regarding the distribution of species at the level of the phylum in the bar plot, *Firmicutes*, *Bacteroidetes*, *Epsilonbacteraeota*, *Proteobacteria*, *Deferribacteres*, and *Actinobacteria* were the representative phyla among the fecal samples of each group. In the DSS (alone) treatment group, *Proteobacteria* and *Deferribacteres* were enriched, while *Firmicutes* were reduced (Figures 6A,B). The proportion of the aforementioned microbiota with changed levels was reversed after acacetin treatment (Figure 6B). On the other hand, the increased abundance of *Bacteroidaceae*, *Deferribacteraceae*, and *Enterobacteriaceae* in the DSS-treated group was reversed at the level of the phylum after acacetin administration (Figures 6A,B).

**FIGURE 7:**
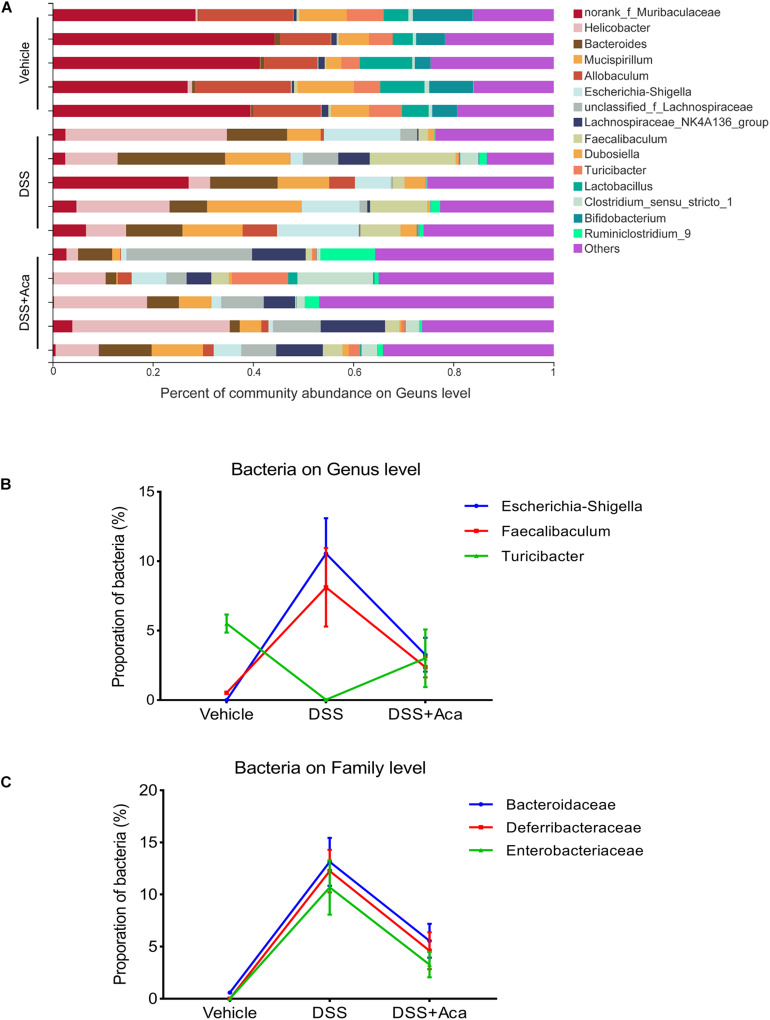
Acacetin regulated the composition and abundance of gut microbiota at the genus level. **(A)** Proportion of dominant genus communities in each group of samples. Less than 5% of the genus communities were merged with others. **(B)** Distribution of the three dominant families (Bacteroidaceae, Deferribacteraceae, and Enterobacteriaceae) in each group. **(C)** Distribution of three dominant genera (*Escherichia-Shigella*, *Faecalibaculum*, and *Turicibacter*) in each group. Data are expressed as the mean ± SEM (*n* = 5 mice per group).

At the genus level, the DSS-treated group exhibited a proportional increase in the abundances of *Escherichia-Shigella* and *Faecalibaculum*, whereas the abundances of those two genera were reduced after acacetin treatment (Figures 7A,C). Furthermore, slight differences in *Family XIII* and *Turicibacter* were noted between the DSS group and the DSS + acacetin group; however, no significant differences were observed (Figures 7B,C).

**FIGURE 8:**
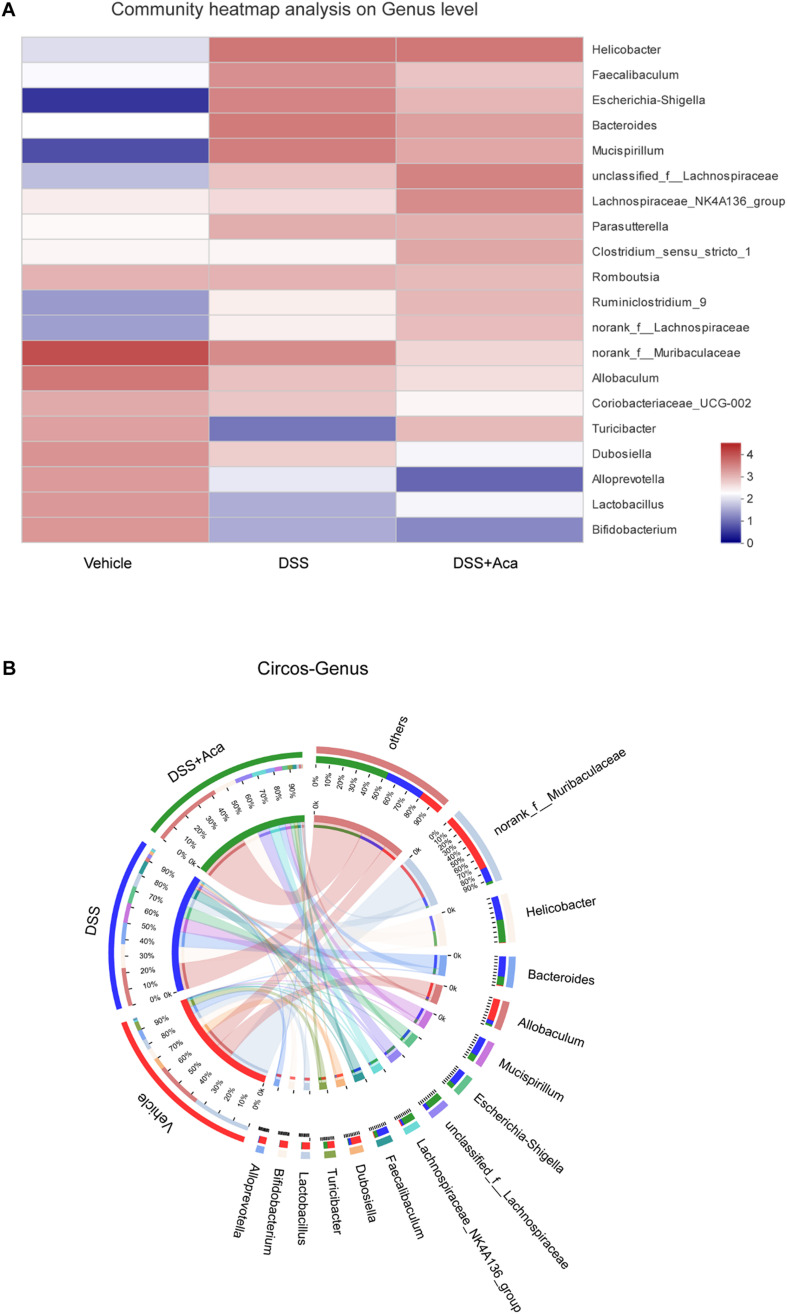
Acacetin regulated the composition and abundance of gut microbiota at the genus level. **(A)** Heatmap comparing expression of different group in 20 genera with a relatively high abundance **(B)** Circos analysis showed the corresponding abundance relationship between different groups and bacterial communities. Data are expressed as the mean ± SEM (*n* = 5 mice per group).

Then we conducted a community heatmap analysis on genus level showing 20 genera with a relatively high abundance (Figure 8A). This result is consistent with the showing of bar plot. Circos analysis confirmed the heatmap results (Figure 8B). *Escherichia-Shigella* was decreased from 76% to 24% and *Faecalibaculum* from 74 to 22% by acacetin treatment (Figure 8B). However, *Turicibacter* was increased, with an abundance of 0.42% in the DSS group and 35% in the DSS + acacetin group (Figure 8B).

These results indicate that DSS-induced colitis could disrupt intestinal microbiota homeostasis, while acacetin treatment could reverse this disruption.

## Discussion

The pathological mechanism of IBD presently remains to be discovered, and IBD has been listed as a modern refractory disease (Ramos and Papadakis, 2019). Conventional therapeutic drugs may produce various side effects, yield a high recurrence rate, and cannot adequately meet the extensive treatment needs of patients with UC. Hence, exploration of the underlying molecular mechanisms and the discovery and development of optimal therapies for IBD are all crucial (Zhang et al., 2014).

To investigate the etiology and pathogenesis of UC, we established a DSS-induced colitis mouse model, which is the most widely used experimental model for UC (Eichele and Kharbanda, 2017). In this animal model, weight loss, bloody diarrhea, and colon length are critical hallmarks by which the severity of colitis can be evaluated. The administration of acacetin was found to improve these symptoms in mice with DSS-induced colitis. Moreover, the mice treated with acacetin alone showed no abnormalities nor toxic effects compared with those treated with DSS throughout the study. These findings suggest that the administration of acacetin at specific concentrations is relatively safe in mice.

Elevated levels of inflammatory cytokines can serve as disease hallmarks in patients with UC (Ishiguro, 1999). Compelling evidence suggests that cytokines are crucial pathophysiological regulators of the occurrence and development of the inflammatory response of IBD (Strober and Fuss, 2011). Interactions between the TNF family of receptors and their ligands are crucial in immune responses (Zhang et al., 2019). The progression of inflammation is associated with TNF-α, as it is involved in the recruitment of leukocytes to the inflamed areas (Gupta et al., 2018). Furthermore, IL-6 plays an important role in the development of inflammation by promoting lymphocyte proliferation, which plays a crucial role in the acute stage of the inflammatory response (Mudter and Neurath, 2007). As a key proinflammatory cytokine, IL-1β can accelerate inflammation and initiate antimicrobial immune responses (LaRock et al., 2016; Monajemi et al., 2018). Both iNOS and COX-2 are key inflammatory mediators involved in the incidence of UC (Cox et al., 2005; Sun L. et al., 2018). In the present study, increased production of iNOS, COX-2, IL-6, TNF-α, and IL-1β was observed in mice with DSS-induced colitis. Oral administration of acacetin at 50 mg/kg significantly reduced the production of these proinflammatory mediators.

Macrophages are crucial for raising appropriate immune response against ingested pathogens. Compelling evidence has shown that macrophages are the main source of inflammatory cytokines during the inflammatory response (Bosma et al., 2016; Jones et al., 2018). Evidence has showed that the reduction of macrophages and inhibition of macrophage response contribute to the therapeutic effect in UC patients (Zeissig et al., 2019). As a colitis model, DSS induces disease symptoms of damaged intestinal epithelial barrier and thereby increases the opportunities that gut lumen contents expose to innate immunity elements (e.g., macrophages) (Eichele and Kharbanda, 2017). Therefore, we further evaluated the anti-inflammatory effects of acacetin *in vitro*. Our results indicate that acacetin can prominently inhibit the levels of NO, iNOS, and COX-2 in LPS-stimulated RAW264.7 murine macrophages, as well as decrease the secretion of NO and the protein expression of iNOS in IFN-γ and LPS induced BMDM cells, which is consistent with the findings of previous reports (Pan et al., 2006). Notably, acacetin led to significant reduction of macrophage infiltration (labeled by F4/80 +), this results suggest that inhibiting macrophage inflammatory response might contribute to the attenuated effects of acacetin on DSS-induced colitis mice.

Moreover, researchers have gained important insights into gut microbiota composition during intestinal inflammation and have revealed new directions for IBD treatment (Ungaro et al., 2017). Several studies have highlighted that the remarkable changes in gut microbiota composition in patients with UC can cause intestinal inflammation. Severe dysbiosis is mainly manifested in the gut of patients with UC, when there is a reduced abundance of *Firmicutes* and *Bacteroidetes*, and increased abundance of *Proteobacteria* (Lepage et al., 2011; Natividad et al., 2015; Munyaka et al., 2016; Glassner et al., 2020).

Some species at the phylum level (e.g., Proteobacteria) could play a role in the pathogenesis of UC, leading to adhesion and invasion of intestinal epithelial cells, disruption of host defense, stimulation of the inflammatory response, alterations in the structure of intestinal microbiota, and ultimately, promote the onset of UC (Bai et al., 2019). Furthermore, *Escherichia*, which is an IgA-coated bacteria at the genus level, is the dominant pathogen involved in the pathogenesis of UC (Xu et al., 2018). *Escherichia-Shigella*, gram-negative bacteria with an outer LPS membrane, can invade the human colonic epithelium and induce inflammatory responses (Gronbach et al., 2014).

In the present study, the abundances of *Proteobacteria* and *Escherichia-Shigella* were reduced following acacetin treatment in mice with DSS-induced colitis. Moreover, significant alterations were observed in DSS-treated mice, as levels of *Firmicutes* were reduced at the phylum level and those of *Escherichia-Shigella* were increased at the genus level; however, acacetin alleviated these changes. Notably, this results are quite consistent with our previously studied natural compounds, including obacunone and pinocembrin (Luo et al., 2020; Yue et al., 2020). There are also some researches indicate that *Escherichia-Shigella* are positively correlated with high expression of inflammatory cytokines, and many studies have shown its adverse effects in IBD (Zeissig et al., 2019). Collectively, our findings provide evidence of the effects of acacetin in reversing the DSS-induced dysbiosis of intestinal microbiota and changes in community composition.

Indeed, the maintenance of the intestinal health depends on a homeostasis between the microbiota and the innate immune system, and macrophages are the most abundant in the innate immune cells (Sica et al., 2015; Yue et al., 2019). Macrophages can generate varieties of inflammatory cytokines, which further activate innate/adaptive immunity, and therefore get rid of the invading bacteria in intestinal lamina propria (Weber et al., 2009). However, excessive inflammatory response can cause the imbalance of immunity homeostasis and influence the microbial composition, which could exacerbate the intestinal inflammation in turn (Sonnenberg and Hepworth, 2019). Moreover, numbers of studies suggested that both exaggerated inflammatory response and dysbacteriosis are often co-existing in IBD patients (Yao et al., 2019). Hence, both inflammatory response and dysbacteriosis are reciprocal causation in the IBD pathogenesis. Our studies showed the evidence that acacetin could ameliorate intestinal inflammation and regulate dysbacteria. There is a need for research to precisely describe the causal link between inflammatory response and dysbacteriosis. Additionally, the precise mechanism of acacetin in the maintenance of intestinal homeostasis needs further clarification.

## Conclusion

Overall, these new findings suggest that acacetin could alleviate DSS-induced colitis in mice by inhibiting the macrophage inflammatory response and reversing gut microbiota dysbiosis.

## Data Availability Statement

The raw data supporting the conclusions of this article will be made available by the authors, without undue reservation.

## Ethics Statement

The animal study was reviewed and approved by the Animal Experiment Ethics Committee at Shanghai University of Traditional Chinese Medicine (Animal license key: PZSHUTCM190315022).

## Author Contributions

JR conducted this study and performed the statistical analysis. BY, HW, BZ, XL, ZY, JZ, and YR assisted with the experiments. JR and BY finished the first draft of the manuscript. WD, ZW, and SM read and critically revised the final version of the manuscript. All authors contributed to the article and approved the submitted version.

## Conflict of Interest

The authors declare that the research was conducted in the absence of any commercial or financial relationships that could be construed as a potential conflict of interest.
