# Supplementary figures and images for: Acacetin Ameliorates Experimental Colitis in Mice via Inhibiting Macrophage Inflammatory Response and Regulating the Composition of Gut Microbiota
Source: Front Physiol. 2021 Jan 18;11:577237. doi: 10.3389/fphys.2020.577237 (PMC7848181; doi:10.3389/fphys.2020.577237)

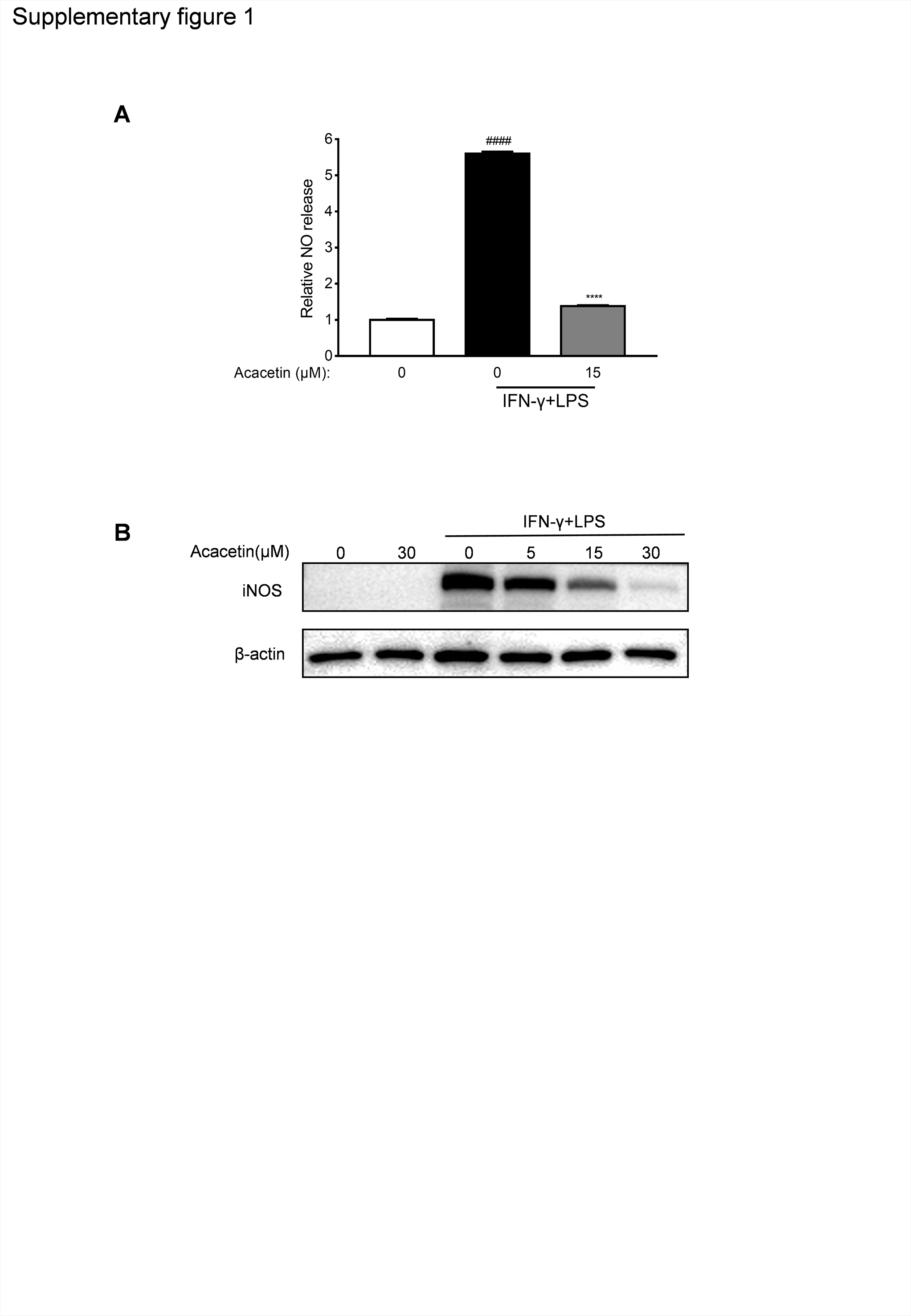

Supplement: Supplementary file 1 [file Image_1.TIF]

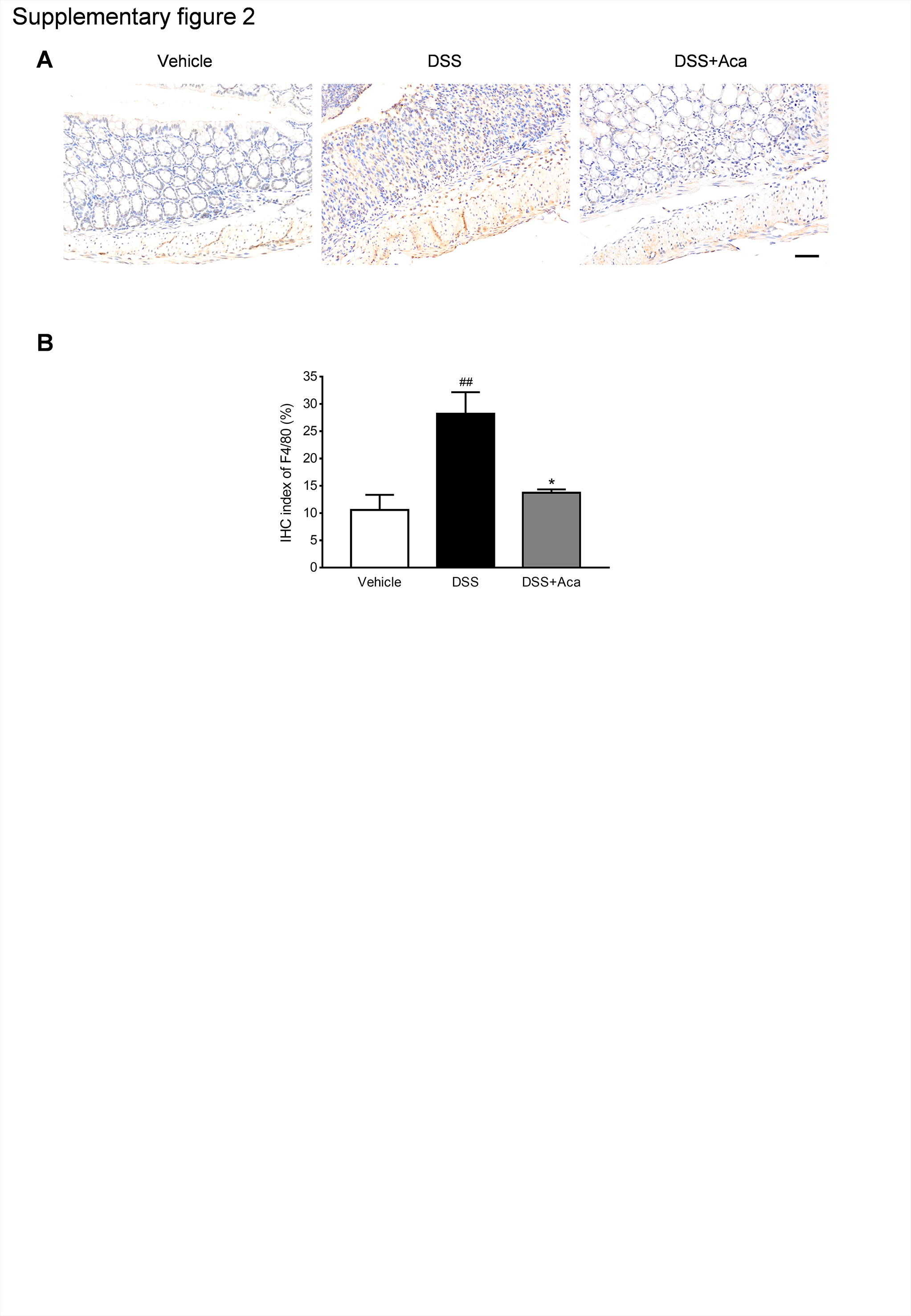

Supplement: Supplementary file 2 [file Image_2.TIF]
